# Metabolic Basis of Creatine in Health and Disease: A Bioinformatics-Assisted Review

**DOI:** 10.3390/nu13041238

**Published:** 2021-04-09

**Authors:** Diego A. Bonilla, Richard B. Kreider, Jeffrey R. Stout, Diego A. Forero, Chad M. Kerksick, Michael D. Roberts, Eric S. Rawson

**Affiliations:** 1 Research Division, Dynamical Business & Science Society–DBSS International SAS, Bogotá 110861, Colombia; 2 Research Group in Biochemistry and Molecular Biology, Universidad Distrital Francisco José de Caldas, Bogotá 110311, Colombia; 3 Research Group in Physical Activity, Sports and Health Sciences (GICAFS), Universidad de Córdoba, Montería 230002, Colombia; 4 kDNA Genomics^®^, Joxe Mari Korta Research Center, University of the Basque Country UPV/EHU, 20018 Donostia-San Sebastián, Spain; 5 Exercise & Sport Nutrition Laboratory, Human Clinical Research Facility, Texas A&M University, College Station, TX 77843, USA; rbkreider@tamu.edu; 6 Physiology of Work and Exercise Response (POWER) Laboratory, Institute of Exercise Physiology and Rehabilitation Science, University of Central Florida, Orlando, FL 32816, USA; jeffrey.stout@ucf.edu; 7 Professional Program in Sport Training, School of Health and Sport Sciences, Fundación Universitaria del Área Andina, Bogotá 111221, Colombia; dforero41@areandina.edu.co; 8 Exercise and Performance Nutrition Laboratory, School of Health Sciences, Lindenwood University, Saint Charles, MO 63301, USA; ckerksick@lindenwood.edu; 9 School of Kinesiology, Auburn University, Auburn, AL 36849, USA; mdr0024@auburn.edu; 10 Edward via College of Osteopathic Medicine, Auburn, AL 36849, USA; 11 Department of Health, Nutrition and Exercise Science, Messiah University, Mechanicsburg, PA 17055, USA; erawson@messiah.edu

**Keywords:** creatine kinase, energy metabolism, cell survival, bioinformatics, systems biology, cellular allostasis, dynamic biosensor

## Abstract

Creatine (Cr) is a ubiquitous molecule that is synthesized mainly in the liver, kidneys, and pancreas. Most of the Cr pool is found in tissues with high-energy demands. Cr enters target cells through a specific symporter called Na^+^/Cl^−^-dependent Cr transporter (CRT). Once within cells, creatine kinase (CK) catalyzes the reversible transphosphorylation reaction between [Mg^2+^:ATP^4−^]^2−^ and Cr to produce phosphocreatine (PCr) and [Mg^2+^:ADP^3−^]^−^. We aimed to perform a comprehensive and bioinformatics-assisted review of the most recent research findings regarding Cr metabolism. Specifically, several public databases, repositories, and bioinformatics tools were utilized for this endeavor. Topics of biological complexity ranging from structural biology to cellular dynamics were addressed herein. In this sense, we sought to address certain pre-specified questions including: (i) What happens when creatine is transported into cells? (ii) How is the CK/PCr system involved in cellular bioenergetics? (iii) How is the CK/PCr system compartmentalized throughout the cell? (iv) What is the role of creatine amongst different tissues? and (v) What is the basis of creatine transport? Under the cellular allostasis paradigm, the CK/PCr system is physiologically essential for life (cell survival, growth, proliferation, differentiation, and migration/motility) by providing an evolutionary advantage for rapid, local, and temporal support of energy- and mechanical-dependent processes. Thus, we suggest the CK/PCr system acts as a dynamic biosensor based on chemo-mechanical energy transduction, which might explain why dysregulation in Cr metabolism contributes to a wide range of diseases besides the mitigating effect that Cr supplementation may have in some of these disease states.

## 1. Introduction

**Figure 1:**
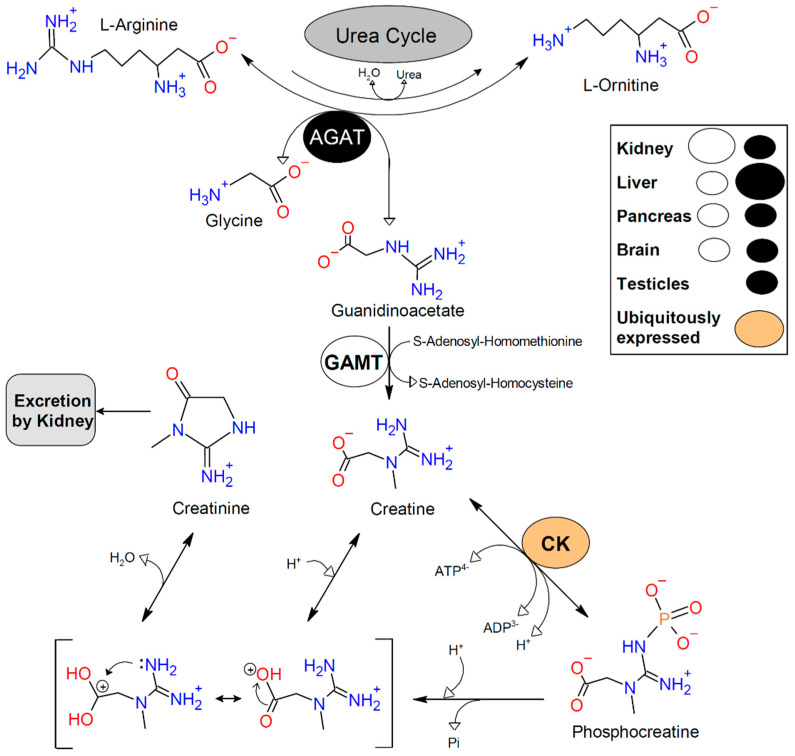
Creatine synthesis/excretion and the creatine kinase reaction. Enzymes are represented by ovals. Once synthesized from L-arginine, glycine, and S-adenosyl-L-methionine, creatine (Cr) is converted to phosphocreatine (PCr) by means of the creatine kinase (CK), which catalyzes the reversible transference of a phosphoryl group (PO_3_^2−^), not a phosphate (PO_4_^3−^), from ATP. The kinetic rate of the non-enzymatic conversion of Cr (or PCr) to creatinine (Crn) depends on the H^+^ concentration of the media. It is important to note that neither Crn nor PCr are substrates of the sodium- and chloride-dependent creatine transporter (not shown). Oval size represents the expression level of AGAT (black), GAMT (white), and CK (orange) in some tissues. For more details related to expression in different tissues or conditions (i.e., pathologies) use the following BioGPS ID numbers: AGAT–2628; GAMT–2593. AGAT: L-Arginine-Glycine amidinotransferase; GAMT: Guanidinoacetate N-Methyltransferase; H^+^: hydrogen ion; Pi: inorganic phosphate. Modified with permission from Bonilla and Moreno [7] using the Freeware ACD/ChemSketch 2021 (Advanced Chemistry Development, Inc., Toronto, ON, Canada).

Creatine (Cr) is a ubiquitous non-protein amino acid (PubChem CID: 586) that is synthesized mainly in the liver, kidneys, and pancreas [1]. However, other tissues (e.g., brain and testes) are also able to produce Cr [2,3,4]. Endogenous Cr synthesis begins with the transfer of the amidino group of L-arginine to the N^α^-amine group of L-glycine following a ping-pong mechanism that is catalyzed by L-Arginine-Glycine amidinotransferase (AGAT-EC 2.1.4.1) [5]. This first reaction yields L-ornithine and guanidinoacetate (GAA), which is then methylated at the original nitrogen of glycine using S-adenosyl-L-methionine as the donor of the methyl group by means of the Guanidinoacetate N-Methyltransferase (GAMT-EC 2.1.1.2). This reaction follows the formation of a strong nucleophile on the deprotonated glycine-derived N of GAA that interacts with the methyl group from the positively charged sulfonium ion of S-adenosyl-L-methionine [6] to produce Cr and S-adenosyl-L-cysteine (Figure 1).

High Cr concentrations are found in skeletal muscle and the brain [8]. High Cr levels are also found in other cells with high energy demands such as the cardiomyocytes, hepatocytes, kidney cells, inner ear cells, enterocytes, spermatozoa, and photoreceptor cells [9,10]. However, approximately 95% of the Cr pool in the body is found in skeletal muscle [11,12,13]. After synthesis, Cr reaches target tissues through the bloodstream, and intracellular transport mediated by a solute carrier protein called sodium- and chloride-dependent creatine transporter (CRT, also known as SLC6A8) [14]. This symporter belongs to a family of neurotransmitter transporters known as solute carrier family 6, which has shown a high affinity to Cr in the plasmalemma (low Km, 15–77 μM) [15,16,17]. Cr is one of the main osmolytes of the central nervous system, which may play important roles in pathophysiological conditions of the brain [18,19]. Currently, some consider Cr a neurotransmitter that may be released in the synapse, re-uptaken by presynaptic CRT, and might either depress post-synaptic GABAergic neurotransmission or stimulate post-synaptic glutamatergic pathways [20]; nevertheless, more studies are needed to generate consensus, in particular by discovering a so far unknown specific post-synaptic Cr receptor [21]. Although some of the aforementioned tissues might synthesize Cr, CRT is necessary to transport endogenous and exogenous Cr to cells with high and fluctuant energy demands for proper physiological function [22].

Cr exists as a zwitterion, with the positive charge on the resonance structures of the guanidinium moiety and the negative charge on the carboxylate oxygen atoms. Thus, it forms a monoclinic crystal system with one water molecule of crystallization [23,24]. These crystals are well-known as creatine monohydrate (CrM), which dehydrates at 110 °C [25]. In Figure 1, the Cr molecule is shown in the zwitterionic form as found in the crystal structure of CrM (where both H-atoms of the water act as hydrogen bond donors—not shown) [23]. It is important to note that the solubility of CrM in water increases with temperature (e.g., 8.5 g·L^−1^ at 4 °C and 14 g·L^−1^ at 25 °C) [26]. It is also notable that CrM has been extensively studied as a nutritional supplement. In this regard, CrM supplementation has been deemed as a safe and effective ingredient across various disciplines ranging from sports nutrition to health and disease [27,28,29,30,31,32,33,34,35,36,37,38,39,40,41,42,43]. Although other forms of Cr have been studied, such as Cr nitrate [44,45,46], there is no evidence that these ingredients are more efficacious relative to CrM [47]. Readers are encouraged to refer to the outstanding invited reviews of this book/special issue on “Creatine Supplementation for Health and Clinical Diseases” to learn more about the effects of CrM supplementation [48].

Cr and its phosphorylated form, phosphocreatine (PCr), have a critical and centralized role in maintaining adenosine triphosphate (ATP) concentrations in tissues with high-energy demands, such as skeletal muscle, heart, and brain [28]. Alterations in Cr concentrations due to CRT, AGAT, or GAMT deficiencies may produce functional changes in these tissues, leading to a wide range of diseases [14,22,49,50,51] that are grouped into the Cr deficiency syndrome [52]. For example, CRT malfunction results in low levels of intracellular Cr, which, while not lethal, induces an impairment in brain energy metabolism to the same extent as deficiencies in the Cr biosynthesis enzymes [22,53]. A dysregulation in Cr metabolism has also been implicated in various pathological conditions including muscle dysfunction, cardiomyopathy, and cancer, among others [48,54]. Given the aforementioned evidence, a systems biology approach is needed to deepen our comprehension of the molecular, cellular, tissue and systemic effects of Cr and its applications to health and disease. Therefore, the aim of this bioinformatics-assisted review was to highlight the most recent findings and up-to-date literature concerning Cr metabolism.

## 2. Methods

To summarize the basis and to report the most recent findings of creatine metabolism, we performed a search of articles indexed in PubMed/MEDLINE, ScienceDirect, Cochrane, SciELO, and Google Scholar databases using terms related to ‘creatine metabolism’. A bioinformatics-assisted analysis was performed for functional annotations within the literature review. To this end, we accessed public databases and repositories such as UniProtKB (https://www.uniprot.org/), PDB (https://www.rcsb.org/), Ensembl (https://www.ensembl.org/index.html), The Gene Ontology Resource (http://geneontology.org/), and the BioGPS–Gene Portal System (http://biogps.org/). Additionally, we used the freely available Search Tool for the Retrieval of Interacting Genes (STRING: https://string-db.org/) to report the experimentally validated interacting proteins. The following options were activated in the STRING tool to obtain the protein–protein interactions network: (i) search—by multiple proteins; (ii) network type—full STRING network; (iii) meaning of network edges—evidence; (iv) minimum required interaction score—high confidence (0.700); and, (v) max number of interactors to show—1st shell = 30, and 2nd shell = no more than 20 interactors. To cluster the most similar nodes of the network into an easily distinguishable function-based classification, we used the Markov Cluster Algorithm for graphs, which is based on simulation of stochastic flow in the obtained graph. The inflation factor was set at 1.5 to balance sensitivity and selectivity. Databases/repositories and bioinformatics tools were accessed from 11 November 2020 to 14 February 2021.

The idea of complexity in biological systems was addressed from a reformulated insight that followed development (self-organizing) to cellular dynamics (functional and structural stability through change–allostasis). Therefore, the retrieved references were summarized and discussed in this review’s narrative to answer certain pre-specified questions: (i) What happens when creatine is transported into cells? (ii) How is the CK/PCr system involved in cellular bioenergetics? (iii) How is the CK/PCr system compartmentalized throughout the cell? (iv) What is the role of creatine amongst different tissues? and (v) What is the basis of creatine transport? 

## 3. Findings

### 3.1. What Happens When Creatine Is Transported into Cells?

Once in the intracellular environment, the creatine kinase (CK, ATP:creatine phosphotransferase, EC 2.7.3.2) catalyzes the reversible transphosphorylation reaction between [Mg^2+^:ATP^4−^]^2−^ and Cr to produce PCr and [Mg^2+^:ADP^3−^]^−^ following a bimolecular nucleophilic substitution reaction [55]. The average concentration of total Cr (free Cr + PCr) in skeletal muscle is around 120 mmoL·kg^−1^ dry mass (≈40 mM) [56] although PCr is found in higher concentration (80–85 mmoL·kg^−1^ dry mass or ≈27 mM, ≈67%) than free Cr (≈40 mmoL·kg^−1^ dry mass or ≈13 mM, ≈33%) [8]. Besides the difference in the free energy change (ΔG°) for the hydrolysis of PCr and ATP at pH 7.0 (−44.58 kJ·moL^−1^ versus −31.8 kJ·moL^−1^, respectively) [57], PCr and Cr are smaller in molecular size, less negatively charged, and more abundant than ATP and adenosine diphosphate (ADP) in cells expressing CK, which represents a thermodynamic and functional improvement to energy metabolism due to a higher intracellular flux of high-energy phosphates [8]. Importantly, in tissues that require large and intermittent amounts of energy, several CK isozymes are ubiquitously expressed in different cellular compartments (e.g., sarcomere, cytosol, mitochondria) connecting places of ATP synthesis with sites of ATP consumption. This is known as the CK/PCr system [11]. 

Cr is spontaneously degraded to creatinine (Crn) in a monomolecular and non-enzymatic reaction that depends on temperature and pH [58]. Crn might diffuse out of the cells to be excreted by the kidneys into the urine with a mean excretion rate of 23.6 mg·kg^−1^·day^−1^ (about 1.7% of the total Cr pool per day) [8]. As more than 90% of Cr and PCr molecules are found in skeletal muscle, Crn excretion is ≈20% less in women and the peak urinary excretion rate is found between 18 to 29 years old [1]. Hence, the daily requirement of Cr from either diet or endogenous synthesis for a 70-kg male is approximately 2 g·day^−1^ [59]. This has raised concerns in vegan and vegetarian population who have been reported to have lower Cr concentrations in different tissues [60,61] since Cr is naturally found in animal products [62,63]. Figure 1 represents the basis of Cr, PCr, and Crn metabolism.

CrM supplementation increases serum and muscular Cr levels [59,64,65], as well as brain Cr levels [66], although no effect is seen with ATP concentrations [67]. While this increase is very significant in serum and skeletal muscle, Cr is not as permeable through the blood-brain barrier as it is in other tissues, so it typically takes higher doses of Cr over a longer periods of time (e.g., 15–20 g per day for 2–4 weeks) to significantly increase Cr content in the brain in healthy individuals [40]. Patients with AGAT and GAMT deficiencies are more dependent on dietary sources of Cr and may need to consume 20–30 g·day^−1^ of CrM habitually to increase and maintain elevations in brain Cr content [68]. For example, in AGAT-deficient patients, it has been shown that after nine months of CrM supplementation (400 mg·kg^−1^·day^−1^), brain Cr levels were increased to 80% of Cr [69]; whereas, GAMT-deficient patients have a slower rise of brain Cr with a nearly complete replenishment after more than two years [70]. Conversely, in response to 20 g CrM in healthy individuals, serum Cr concentration increases by 50-fold (peak value of serum Cr is approximately 2.17 ± 0.66 mM) 2.5 h following ingestion [71]. However, in response to lower doses (≈2 g CrM), Cr increase in blood is less significant [72]. In skeletal muscle, total Cr levels increase by about 25% after CrM supplementation, while increases up to 37% occur if the ingestion is accompanied with exercise training [1]. It has been reported that CrM supplementation increases muscle PCr content by ≈20%, generally from 80 to 95 mmoL·kg^−1^ dry mass [64,65]. Brault et al. (2007) demonstrated that CrM does not alter the PCr/total Cr ratio and hence the ΔG° for the hydrolysis of ATP at rest. The authors reported a linear increase of PCr and total Cr concentrations in the *vastus lateralis* after five days of CrM supplementation (0.43 g·kg body mass^−1^·day^−1^) using ^31^P and ^1^H magnetic resonance spectroscopy [73]. This increase in muscle PCr concentration and the maintenance of the PCr/total Cr ratio are critical in regulating the skeletal muscle bioenergetics due to the crucial role of the CK/PCr system [74]. It is well-established that PCr concentration and oxygen uptake (VO_2_) vary with similar kinetic profiles from the start-up of the exercise until a new state of energy production by oxidative metabolism [74,75], which has been explained as a function of the mitochondrial resistance and the metabolic capacitance of the CK reaction [76,77]. The regulation of mitochondrial respiration is intimately linked to the CK/PCr system, where changes in the time constant (τ) for the decrease in muscle PCr concentration become critical, as it has been shown in both the “electrical” [78] and “hydraulic” [79] analog models of oxidative metabolism. In fact, recent findings have reinforced the notion that the decline in mitochondrial function due to the aging process is closely related to the muscular performance (i.e., post-exercise PCr recovery rate) [80]. In accordance with these models, an increase in the muscle metabolic capacitance (determined by the augmentation in total Cr) after five days of CrM supplementation (20 g per day) has resulted in a longer τ (slower PCr kinetics) [81], and a slower VO_2_ response [82]. Thus, the rise in PCr levels following the CrM supplementation optimizes the cellular thermokinetics of energy transduction by regulating the cellular ATP/ADP ratio [7].

### 3.2. How Is the CK/PCr System Involved in Cellular Bioenergetics?

Cell growth and survival depend on constant ATP regeneration in order to sustain motor proteins (e.g., muscle contraction, vesicle trafficking), ion pumping, protoplasmic streaming, cytoskeletal rearrangement, among others. ATP is synthesized either through substrate-level phosphorylation or through oxidative phosphorylation [83]; however, to guarantee it is mostly used in contraction machinery, ATPase pumps, and other organelles (i.e., nucleus, endoplasmic reticulum, etc.), the cell relies on a phosphotransfer network that is based on the CK/PCr system [84]. This system encompasses two cytoplasmic and two mitochondrial CK (MtCK) isozymes. MtCK is functionally associated with oxidative phosphorylation by co-localization with the adenine nucleotide translocase (ANT, also known as SLC25A4), and by the formation of a proteolipid complex (physical interaction) with the voltage-dependent anion channel (VDAC) and other biological structures in the mitochondrial inner membrane (e.g., cardiolipin-rich domains and other anionic phospholipids) [85]. This system allows ATP to be generated in mitochondria, and this ATP can be subsequently utilized by MtCK to synthesize PCr. This newly-synthesized PCr can then be transported to the cytosol where isozymes of CK resynthesize ATP from ADP [86]. Bessman and Carpenter (1985) initially called such transfer of high-energy phosphates the Cr-PCr shuttle [87]. Thus, in cells that require constant energy for metabolic reactions, PCr acts as an abundant energy buffering molecule that facilitates phosphotransfer reactions by CK parallel to ATP diffusion. Because of fluctuating energy requirements in skeletal muscle and other tissues, the CK/PCr system not only serves as “spatial” but also “temporal” energy buffer where PCr follows closely the energy-requiring processes (e.g., force generation throughout the contraction cycle) while ATP remains constant [88]. The CK/PCr system also improves the thermodynamic efficiency of ATP hydrolysis by maintaining low intracellular ADP concentration and a high ATP/ADP ratio at those subcellular sites where CK is functionally coupled to ATP-requiring processes [13]. In this sense, the CK/PCr system’s ATP generation capacity is quite high and exceeds both ATP utilization and ATP resynthesis by other energy-producing pathways (e.g., oxidative phosphorylation and glycolysis) [89,90]. For example, the maximal rate of ATP synthesis by the CK reaction in rat cardiac muscle is 30 µmoL·s^−1^·g^−1^, which is much higher than ATP synthesis by oxidative phosphorylation (2.5 µmoL·s^−1^·g^−1^) or by de novo pathways (0.39 µmoL·s^−1^·g^−1^) [11]. This small reduction in net energy balance (work done per hydrolyzed ATP) makes CK system become crucial for survival, from an evolutionary point of view; in fact, these phosphagen kinase systems date back to several hundred millions years to early metazoan and bacteria [91,92].

#### CK Isozymes

As mentioned before, the CK isozymes are the core of the CK/PCr system during the process of energy transduction in tissues with high and intermittent energy demands (i.e., skeletal muscle, brain, heart, etc.). Cytosolic CK may be assembled as a protein hetero- or homodimer after binding the M-CK and B-CK subunits to form the MM-, MB-, and BB-CK isozymes, which have an approximate relative mass of 80,000–86,000 [93]. MM- is the major isoform in muscle and heart, MB- is mainly present in the myocardium, and BB-CK exists in many tissues, especially the brain. In skeletal muscle, besides being specifically located at the sarcomeric M-band, a significant proportion of MM-CK is in close proximity to the sarcoendoplasmic reticulum Ca^2+^-ATPase (SERCA) and sarcolemma. This guarantees the thermodynamic efficiency of ATP hydrolysis (ΔG° is kept high) [94]. Interestingly, Ramírez et al. (2014) have reported specific phosphorylation of the B-CK isoform at Ser6 can be facilitated by different AMP-activated protein kinase (AMPK) isoforms [95]. This does not affect enzymatic activity, but causes its localization to specific subcellular compartments (e.g., endoplasmic reticulum) as well as its co-localization with the highly energy-demanding SERCA. Moreover, it has been shown that a decrease in intracellular pH in muscle activates MM-CK to facilitate ATP regeneration [96], which might be expected after heightened muscle activity if we consider the optimum pH of this enzyme is between 6.5 and 6.7 [97].

There are two mitochondrial CK isoenzymes: the striated muscle specific, sarcomeric MtCK or sMtCK and the ubiquitous MtCK or uMtCK [11]. Although there is a high degree of sequence homology between these two, sMtCK and uMtCK differ in many biochemical and biophysical parameters. For example, in comparison to M- and B-CK isoenzymes, which are protein dimers, sMtCK and uMtCK are homooctamers (relative mass of ≈340,000) composed of four dimers as the stable building blocks [98,99]. The MtCK is localized between inner and outer mitochondrial membranes in co-localization with ANT, but is also anchored to the cytoskeleton via VDAC and the mitochondrial interactosome [100]. The different characteristics and expression patterns of the CK isozymes account for the cell-compartmentalized and tissue-specialized functions as might be expected (Table 1).

### 3.3. How Is the CK/PCr System Compartmentalized throughout the Cell?

#### 3.3.1. Mitochondrial Reticulum

Energy-demanding cells have a high hydrolase activity (e.g., ATPases) throughout the entire protoplasm and membranes. The purpose of this is to release the chemical energy stored in the covalent bonds of phosphagen compounds and thereby cover the requirements for survival and growth. At that point, cellular organelles should not be viewed as isolated compartments but, instead, should be seen as a super-connected network of subsystems that maintain cellular allostasis. In this regard, the mitochondrial reticulum has been proposed to exist as a conductive pathway for energy distribution, based on energy distribution across the cell via a much more rapid direct electrical conduction of the mitochondrial membrane potential [102] and constant metabolite diffusion [103]. As a conductive network for skeletal muscle energy distribution, the mitochondrial reticulum helps to cover more surface area and minimize distances for metabolites to support the rapid energy transduction over large cell regions. This connectivity puts the energy distribution system at risk though, because damaged elements could compromise the entire network. Nevertheless, it has been shown that several intermitochondrial junctions exist, which limits the cellular impact of localized dysfunction. However, the dynamic disconnection of damaged mitochondria allows the remaining mitochondria to resume normal function within seconds [104]. In this context, wherever the mitochondrial reticulum is extending, MtCK and PCr are likely present to support energy transduction between metabolic microcompartments [103].

Octameric MtCK has membrane-binding properties, and it acts as a typical peripheral membrane protein. More specifically, it is anchored to cristae and the peripheral intermembrane space of mitochondria, showing a high affinity for acidic phospholipids, especially cardiolipin (diphosphatidylglycerol) in the inner membrane, and to VDAC in the outer membrane [11]. Hence, because of its size and its binding properties, MtCK can bridge the intermembrane space [105]. As previously mentioned, there is also enough evidence to suggest that MtCK is functionally close to the transmembrane ANT in the inner mitochondrial membrane [85]. This proteolipid complex comprising ANT, ATP synthasome, MtCK, VDAC, membrane phospholipid compounds, and β-tubulin in cytoskeleton contact sites has been named as mitochondrial interactosome and is an important regulator of mitochondrial oxidative metabolism [106]. It has been shown that endogenous ADP is a crucial regulator of oxidative phosphorylation but only in the presence of Cr and MtCK, which is strongly amplified by the co-localization with ANT due to the continuous recycling of adenine nucleotides within the mitochondrial interactosome [107]. The MtCK transfers the phosphoryl group from mitochondrial ATP to Cr producing PCr and recycling ADP in mitochondria. Recycled ADP is returned to F_o_F_1_-ATP synthase complex due to its functional coupling with MtCK while PCr leaves mitochondria due to the high selective permeability of VDAC for this compound [100]. The remarkably high affinity of MtCK for both Cr and PCr, and the metabolic channeling of ATP and ADP via ANT, show that PCr is the main carrier for energy flux carried out from mitochondria reticulum [108]. To highlight, Karo et al. [109] developed a coarse-grained model to simulate the molecular dynamics of the MtCK system, including MtCK, transmembrane ANT, and a membrane composed of phosphatidylcholine, phosphatidylethanolamine, and cardiolipin (2:1:1). The model was validated against many structural and dynamical experimental properties, which makes it useful for future developments. For a recent and comprehensive review of the molecular characteristics and essentials of the mitochondrial proteolipid complexes of CK please refer to Schlattner et al. [85].

Recent studies have proposed that Cr metabolism might have a potential role in thermogenesis. This heat production process occurs in mitochondria through the uncoupling proteins (UCPs), which serve as H^+^ carriers from intermembrane space to matrix and thereby shunt energy from electron transport chain during ATP synthesis [110]. In general, this process releases the oxidation energy as heat and decreases ATP synthesis rates. Initially called thermogenin, UCPs belong to the solute carrier family 25 (SLC25), with UCP1 (also known as SLC25A7) as the isoform only expressed in the brown adipose tissue (BAT) [111]. Notwithstanding, several UCP isoforms have been reported in humans. UCP2 (SLC25A48) is expressed in various tissues, such as skin, muscle, pancreas, adipose tissue [112]. UCP3 (SLC25A9) is mainly found in cardiac and skeletal muscle, and UCP4 (SLC25A27) and UCP5 (SLC25A14, also called brain mitochondrial carrier protein-1) are expressed in the central nervous system [113]. Although these UCP isoforms have high homology and structural similarities (i.e., C- and N-terminal chains are found towards the intermembrane space) [114,115], their biological role and the H^+^ transport mechanism seem to be different according to the cell/tissue where they are expressed [116]. After stimulation and in presence of fatty acids, UCPs allow the passive movement of H^+^ from intermembrane space to mitochondrial matrix via two putative mechanistic models including: (i) the fatty acid cycling model, which is based on a “flip-flop” mechanism, where the UCPs can also transport anions (e.g., fatty acids derivatives) outside the intermembrane space in order to allow them to protonate and get back to matrix [117,118]; and, (ii) the fatty acid buffering model, in which UCPs are proton carriers with fatty acids working as co-factors that interact with carboxyl groups of negatively charged amino acids to mediate the H^+^ transport through a hypothetic channel [119]. An alternative modification of the latter model is called the fatty acid shuttling model, where the fatty acid anions bind inside the UCP cavity resulting in a conformational change that shuttles the H^+^ [120]. Taking into account differences in molecular mechanisms among isoforms, UCPs possess negative regulation sites for nucleotides (ADP, GDP, etc.) and Pi, which can bind to the cavity and allosterically displace fatty acids from the peripheral site and consequently prevent H^+^ transport [116,121]. Therefore, it is plausible that the metabolism of high-energy phosphates regulates this mitochondrial energy dissipation. 

Interestingly, CK activity and genes related to Cr metabolism are coordinately elevated by cold-exposure in beige/brite adipocytes [122]. Additionally, according to Kazak et al. [123] the genetic-induced depletion of Cr in mice significantly blunts β3-adrenergic activation and affects whole-body oxygen consumption. These authors also reported an obese phenotype in mice lacking the capability of the adipose tissue to synthesize Cr, and Cr supplementation rescues aspects of thermogenesis in these animals. Bertholet et al. [124] implemented patch-clamp and bioenergetics analyses to characterize wild-type and *UCP1*-negative beige/brite adipocytes from C57BL/6J mice. These authors found that UCP1 appeared non-essential for the process of browning (because robust mitochondrial biogenesis was still observed in cells lacking UCP1 expression), as well as higher *CKMT2* expression in the *UCP1*-negative model, which supported Cr cycling as a UCP1-independent thermogenic mechanism. Since *UCP1*-negative adipocytes are unable to exhibit a rapid adaptive thermogenic response [123], the ATP-dependent thermogenic pathways may play a key role in diet-induced thermogenesis [125]. Nowadays, it is hypothesized that Cr metabolism may also provide an alternative mechanism of heat production following a futile cycle [126] (also called Cr-driven thermogenesis or Cr-dependent substrate cycling [127]) that coexists with the ATP-dependent Ca^2+^ cycling by SERCA as the main UCP1-independent thermogenic pathways in BAT and beige adipocytes [128]. While the existence of a novel mitochondrial phosphocreatine phosphatase has been hypothesized to explain this highly unusual type of Cr utilization in thermogenic adipocytes [126,128], Wallimann et al. [129] proposed that Cr may operate as part of the classical CK/PCr system by providing ATP to other thermogenic pathways, such as the previously mentioned ATP-dependent Ca^2+^ cycling by SERCA. In spite of these findings, a recent study by Connell et al. [130] showed that CrM supplementation (20 g·day^−1^ for nine consecutive days) did not enhance BAT activation after acute cold exposure in young, healthy, lean, and vegetarian adults. Thus, future clinical research is needed to determine if Cr metabolism plays a role in beige/brite adipose tissue thermogenesis.

#### 3.3.2. Cytosol and Cytoskeleton

In the cytosol, CK is functionally coupled to the enzymatic machinery of glycogenolysis and glycolysis to form an efficient subsystem of energy production and transduction [131]. Several proteins of the glycolytic machinery are located at the I-band and associated with the thin filaments in the sarcomere. Similarly, most of the soluble MM-CK is located at I-band, and, thus, serves to maintain the efficiency of the extramitochondrial ATP production [11]. During periods of high energy demand, the net result of the CK reaction includes the breakdown of PCr to Cr and Pi while ATP and ADP concentrations remain almost constant [132]. This net release of Pi is a seldom-recognized consequence of the CK reaction and is proportional to the amount of PCr hydrolyzed [13]. In this sense, besides buffering ATP concentrations, the CK/PCr system also provides a source of increasing Pi with elevations in work rate [133]. The reaction has a regulatory effect on glycogenolysis and glycolysis since Pi can stimulate glycogen phosphorylase and phosphofructokinase [13]. In fact, anchoring of MM-CK to the I-band via phosphofructokinase has been shown to be strongly pH-dependent and taking place below pH 7.0 [131]. It is important to note that several glycolytic enzymes, glycogen phosphorylase, CK, and adenylate kinase, bound to phosphofructokinase [134], as a key enzymatic complex to regulate glycolysis [135]. Moreover, M-CK has also been shown to bind β-enolase as an anchor for glycolytic complexes on the sarcomere [136]. 

Overall, while mt-CK activity lowers cytosolic Pi levels, cytosolic CK isozymes have the opposite effect [137]. This not only supports the notion that CK/PCr system acts as an important regulator of mitochondrial ATP synthesis with Pi as a primary controller of oxidative phosphorylation [138] but also demonstrates its interconnectivity with glycolysis. According to the molecular system bioenergetics-part of the systems biology approach [139], in vivo regulation of cellular respiration and energy fluxes (i.e., system level properties) depend on intracellular interactions between mitochondrial reticulum, cytoskeleton, intracellular ATPases, and cytoplasmic glycolytic machinery (i.e., system’s components) [140]. For example, hexokinase and β-tubulin (important proteins for glycolysis and cytoskeleton modulation, respectively) have been shown to regulate the mitochondrial outer membrane permeability via interaction with VDAC within the large intermembrane protein supercomplex of the mitochondrial interactosome [141]. 

Hexokinase binds to VDAC to regulate mitochondrial function while stimulating glycolysis considering that ATP from oxidative phosphorylation will be guided directly to active sites of the glycolytic machinery (like hexokinase-2) [142]. In cancer cells, this functional and structural proximity leads to a common metabolic phenotype where there is a higher glycolysis rate rather than oxidative metabolism for energy production, known as the “Warburg effect” [143]. Besides the direct antioxidant properties [144], the potential anti-tumor progression that has been associated to Cr and cyclocreatine administration [126] might be partially explained by a less glycolytic rate in tumor cells. Based on the Warburg hypothesis, it has also been discussed that high-intensity exercise may inhibit glycolysis and have a stronger anti-tumor growth effect in comparison to moderate-intensity exercise [145]. Since immune-based manipulation of glucose metabolism are a subject of high interest to ameliorate cancer progression [146,147], further research might evaluate the effects and regulation of high-intensity exercise plus CrM supplementation (and derivatives) on tumor growth. Several authors have reported lower lactate accumulation after Cr administration in different conditions both in vivo (human and animal models) and in vitro studies [148,149,150,151,152,153]. This reduction in lactate concentration, especially during circumstances requiring high amounts of ATP, has been attributed to less reliance on glycolytic ATP production due to higher intracellular PCr levels after Cr administration. Interestingly, PCr not only inhibits phosphofructokinase [154] and pyruvate kinase [155] activity, but this molecule also stimulates fructose-1,6-biphosphatase [156]. The enzymatic regulation and the frequent rest lapses of intermittent exercise (that contribute to the maintenance of ATP, PCr, and malate levels) may consequently inhibit glycolysis. Although the exact mechanism is still unknown, PCr has also been proposed to modulate AMPK by regulating intracellular PCr concentration. Ponticos et al. [157] reported in vitro that an increase in the intramuscular concentrations of PCr inhibits AMPK activity while free Cr antagonizes this inhibition. A decrease in the AMP/ATP ratio also inhibits this metabolic regulator [158]. Because AMPK activation occurs in response to a reduction in energy availability, an increase in the energy availability by optimization of the phosphagen system after Cr supplementation would favor a direct inhibition and/or delay of AMPK activation during periods of high-energy demand. Recently, Zhang et al. [159] showed that dietary addition of CrM (1200 mg·kg^−1^) inhibited the AMPKα pathway and reduced muscle glycolysis, which improved meat quality in transport-stressed broilers. In spite of the above, Taylor et al. [160] found that PCr neither inhibited phosphorylation of AMPK by LKB1 (AMPKK), nor inhibited recombinant or highly purified rat liver AMPK. Moreover, Eijnde et al. [161] reported that CrM supplementation during two weeks of immobilization (15 g·day^−1^) and subsequent six-week rehabilitation training (2.5 g·day^−1^) did not affect the expression of AMPK α1, α2, or β2 subunits or the phosphorylation status of AMPK α1. Thus, while certain evidence suggests that changes in PCr concentrations might regulate AMPK activity, other studies do not support these findings. Therefore, future studies are needed to better comprehend the mechanisms by which CrM supplementation modulates glycolysis at high work rates as well as AMPK activity. 

Cr metabolism may also regulate cellular processes by being involved with cytoskeletal dynamics. Aside from serving as a scaffold to maintain cellular integrity by cross-linking microtubules (tubulin), microfilaments (actin) and intermediate filaments (lamin), the cytoskeleton possesses architectural, mechanical, and signaling functions that connect cellular subsystems (e.g., sarcomere) to other organelles (e.g., mitochondrial reticulum, membrane and nucleus) [162]. In this regard, it has been shown that the interaction between cytoskeletal proteins and mitochondria (e.g., β-tubulin-VDAC interaction) modulates cellular energy metabolism by contributing to the switch from oxidative phosphorylation to glycolysis [163]. The proteins of the mitochondrial interactosome, including the MtCK, are responsible for this regulation [164]. Furthermore, in myocytes, the Four-and-a-Half Lim 2 (FHL2) not only binds to titin and serves as an important mechanosensor that triggers hypertrophy in response to strain (via mitogen-activated protein kinases, MAPKs) but also docks key metabolic enzymes involved in the energy transduction process like M-CK, adenylate kinase, and phosphofructokinase [165]. Refer to Henderson et al. [166] for a comprehensive review regarding cytoskeleton architecture and proteins functions. Maintaining a close interaction between mitochondrial reticulum and myofibrils through a highly structured cytoarchitecture seems critical for optimal energetic regulation, especially by compartmentalized phosphotransfer enzymes and glycolytic machinery [167]. Hence, energetic interactions between subcellular organelles in high-energy demanding cells depend largely on phosphotransfer kinases, the most important being CK, and their connections to cytoskeleton proteins [168]. It is not surprising that energy disturbances due to the dysfunction of mitochondria and mitochondria-cytoskeleton connections/interactions can lead to various congenital and age-associated diseases [169,170,171,172,173]. 

The extensive cytoskeletal reorganization that occurs before and during cell fusion (e.g., myoblast fusion during muscle development) is highly dependent on ATP hydrolysis, and the polymerization and dissociation of actin monomers may require up to 50% of cellular energy expenditure [174]. As an ATP-consuming process, actin cytoskeleton polymerization can be also optimized by higher phosphagen availability. This was demonstrated by O’Connor et al. (2008) by assessing the in vitro and in vivo effects of Cr administration on myoblast fusion. The authors concluded that Cr enhanced both myotube growth and myonuclear addition in a CK- and actin polymerization-dependent manner [175]. Current available evidence also suggest that ATP produced by cytosolic CK isoforms near the ends of myotubes plays a key role in myoblast fusion during myogenesis [176,177].

#### 3.3.3. Nucleus

The role of the cytoskeleton is not limited to maintaining the structural integrity of the cell, but is also closely involved in gene expression. The linker of nucleoskeleton and cytoskeleton (LINC) complex has been described as an important system of proteins that provides structural support to maintain the nuclear morphology and genome integrity by means of the interaction between the nucleoskeleton with the cytoskeleton [178]. Also, the LINC complex regulates dynamic events including DNA replication and gene transcription [179], and miRNA processing [180]. Briefly, the LINC complex contains three proteins: (i) lamins, which are the basic subunit of intermediate filaments as previously mentioned; (ii) SUN domain proteins, which correspond to Sad1 and UNC-84 proteins; and, (iii) nuclear envelope spectrin repeat proteins (nesprins) [181]. Here, various FHL isoforms (mainly FHL1) have been reported to interact with different transcription factors in the nucleus (e.g., NFAT proteins or RBP-J) that are involved in cell proliferation and differentiation, as well as with the pro-apoptotic protein Siva where it is involved in cell survival [182]. 

Nuclear migration is seemingly critical for muscle development, fertilization, neuronal development, and cellular polarization, with the ATP-binding protein known as torsinA as the main candidate that mediates these processes [183]. It has been identified that the ATPase activation of torsinA involves two stimulatory co-factors, LAP1 and LULL1 [183]. Accordingly, DNA replication, chromatin remodeling, gene transcription and active transport of macromolecules across the nuclear envelope are highly dependent upon constant ATP generation [86]. While principles governing nuclear energetics and energy support for nucleocytoplasmic communication are still poorly understood, it has been demonstrated that mitochondrial ATP production is required to support energy-consuming processes at the nuclear envelope, while glycolysis by itself might be insufficient to perform such a function [184]. In addition, inhibition of nuclear transport by disruption of the adenylate kinase might be rescued through upregulation of alternative phosphotransfer pathways, such as the CK/PCr system, underscoring the plasticity of the cellular energetic network [185]. For instance, nucleoside-diphosphate kinase (NDPK), which is localized in mitochondria, cytosol, and nucleus, is in charge of nucleoside triphosphates synthesis other than ATP [186]. The γ-phosphate of the ATP molecule is transferred to the β-phosphate of NDP via a ping-pong mechanism, using a phosphorylated active-site intermediate [187]. In addition, NDPK possesses several enzymatic activities, acting as serine/threonine-specific protein kinase, geranyl and farnesyl pyrophosphate kinase, histidine protein kinase, and 3′-5′ exonuclease (UniprotKB ID: P15531). Therefore, NDPK facilitates channeling nucleoside triphosphates into protein synthesis/DNA replication complexes, and GTP/GDP exchange on Ran GTPase as an essential factor in nuclear transport through importins and exportins [188]. Particularly, CK is essential for energy distribution in the nucleus because of its buffering ATP concentrations. Thus, the interaction between these systems (adenylate kinase, CK, and NDPK) secure proper nucleotide ratios at and across the nuclear envelope, sustaining the high energy demand of ATP and GTP hydrolysis [86].

#### 3.3.4. Ion Pumps

MM-CK is functionally coupled to SERCA to favor Ca^2+^ handling (optimal uptake rate and sarcoendoplasmic reticulum content) [189]. Despite the presence of high levels of cytosolic ATP, depletion of PCr impairs Ca^2+^ uptake [190]. This clearly shows the importance of MM-CK in rapid rephosphorylation of local ADP produced in the SERCA reaction, independently from the cytoplasmic environment, demonstrating that bound MM-CK acts in a non-equilibrium manner [94]. On the other hand, co-localization and/or functional coupling of CK isoforms with the Na^+^/K^+^-ATPase [191,192], the ATP-gated K^+^-channel [13], the H^+^/K^+^-ATPase [191] and the Na^+^/Ca^2+^ exchanger [193] have been reported in different tissues.

#### 3.3.5. Motor Proteins

Cellular processes involving contractile machinery for cell division and fusion (e.g., satellite cell proliferation and myoblast fusion, respectively), cell motility (e.g., sperm motility), organelle and cytoskeletal rearrangement (e.g., morphology remodeling after virus infection), membrane transport and clathrin-mediated vesicular trafficking (e.g., GLUT4 endo- and exocytosis), and signaling transduction (e.g., the MAPK pathway c-Jun NH_2_-terminal kinase [JNK]) rely vastly on motor proteins. These large mechanochemical ATPases traverse the cytoskeleton by producing a force that propels them and their cargo forward by transforming chemical energy into mechanical movement via ATP hydrolysis [194]. There are three classes of motor proteins: (i) myosin isoforms, dyneins, and kinesins. Approximately 40 isoforms have been reported in humans, and these proteins traverse on actin filaments to translocate their cargo via anterograde transport (i.e., outward movement from the cell body toward the axon or the cell membrane). Various myosin isoforms are involved with muscle movement, cytokinesis, and transporting cargo along microfilaments [195]. Dyneins traverse cargo on microtubules mostly via retrograde transport (i.e., towards the cell center). Sixteen mammalian classes of these motor proteins exist, and can be divided into cytoplasmic dyneins (vesicle trafficking) and axonemal dyneins (movement of cilia or flagella) [196]. Kinesins usually traverse anterogradely on microtubules, and are in charge of transporting cargos such as vesicles, organelles, mRNA, proteins, and chromosomes (14 classes have been described) [197].

Motor proteins act by hydrolyzing ATP, which results in conformational changes that propel them and the cargo towards its destination. Given the high amounts of ATP involved in these processes, it is logical to link the CK/PCr system to these mechanical processes. The roles and importance of M- and B-CK in different tissues have been well-described [11,13,198]. As mentioned previously, MM-CK is bound to M-line and some relevant proportions of this isozyme are in the I-bands of sarcomeres. This position of the MM-CK is crucial for maintaining the efficiency of ATP regeneration in actomyosin ATPases during muscle contraction. Conversely, PCr accelerates the muscle relaxation from rigor tension by decreasing the necessary ATP concentration possibly due to co-localization of M-CK and the very rapid ADP rephosphorylation [199]. On the other hand, various myosin-associated motor mechanisms involved in the formation of the specialized structures at the phagosome may also be B-CK dependent (i.e., B-CK co-localizes transitorily with F-actin at the nascent phagosome), given that actin polymerization and particle adhesion are highly controlled by the ATP/ADP ratio [200]. It is important to note that cytoskeletal regulators of myofibrillogenesis, rearrangement of mitochondrial reticulum, intracellular signaling, and gene expression, such as desmin, can interact with actin, tubulin, plectin (cytolinker protein), and dynein to facilitate these biological processes [169]. In other cells (e.g., astrocytes and fibroblasts), B-CK facilitates actin-driven cell spreading and migration by localizing in peripheral cellular structures [201]. Indeed, animal models deficient in B-CK, M-CK, or Cr have shown a significant decline in brain, muscle, heart, and sensory organs function. These models have been critical to study how disturbances in Cr metabolism affects various tissues and/or involved with certain disease states [2,15,22,198,202].

**Figure 2:**
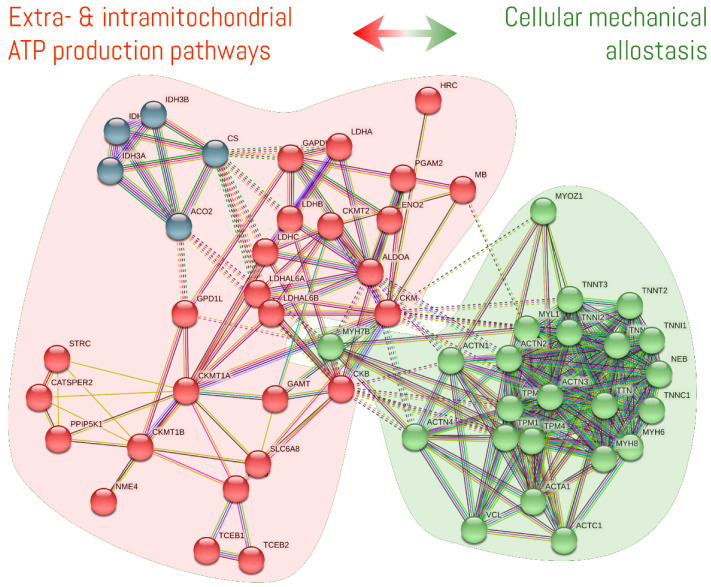
Clustering of CK-interacting proteins using the Markov Cluster Algorithm. Network nodes represent proteins while edges represent protein–protein associations. The red cluster includes a subgroup of enzymes participating in the tricarboxylic acid cycle that are represented in the graph with blue nodes. To visualize our interactive network access to this permanent link: https://version-11-0b.string-db.org/cgi/network?networkId=bu20zAE45PpB (accessed on 14 February 2021).

Hu et al. [203] examined protein–protein interactions using several experimental databases to describe CK-associated networks in *homo sapiens*. In short, these authors reported more than 120 proteins interacted with B-CK, and approximately 90 proteins interacted with M-CK. The identification of NFKB1, FHL2, MYOC, and ASB9 as hub proteins associated with CK further suggest an important interaction with cytoskeletal- and motor-related proteins. NFKB1 is a functionally cytoskeleton-dependent protein while FHL2 was already described as an important scaffold protein involved in mechanosensing and glycolysis. MYOC is a motor protein classified as class-I myosin, and ASB9 is a protein involved in the ubiquitination-mediated proteolysis pathway. To group the most relevant and recent CK-interacting proteins into an easily distinguishable classification based on function, we submitted various CK isoforms (CKMT1B, CKM, CKB, CKMT2, and CKMT1A) to STRING. Subsequently, we performed a clustering analysis using the Markov Cluster Algorithm for graphs. As shown in Figure 2, two main clusters were identified through this bioinformatics analysis. One cluster of proteins is enriched with enzymes involved in extra- and intramitochondrial ATP production. The second cluster contains proteins that are involved in cellular mechanical allostasis such as cytoskeletal and contractile machinery.

Intriguingly, the results of our clustering analysis of CK-interacting proteins highly agree with the contention suggesting cellular allostasis is regulated through a complex balance of subcellular energy production and cellular mechanics. This highlights the critical role of force-sensitive cytoskeleton [204]. In this sense, the CK/PCr system could be viewed as a dynamic biosensor of cellular allostasis, and this may explain various positive benefits of CrM supplementation. On this basis, a biosensor is a system composed by a receptor (that interacts with the environment) and a transducer (that converts the biological response into an energy signal) to elicit a physiologically relevant function [205]. The CK/PCr system encompasses a molecular network made of enzymes and metabolites capable of sensing multi-input physiological changes to produce a broad spectrum of specific energy signals (e.g., chemical, electric, mechanical, heat) with biological significance (e.g., muscle contraction, cell motility, human vision, thermogenesis). The CK/PCr system is dynamic in nature but can also operate within adjustable ranges and sensitivities based on the potential alterations in Cr and PCr concentrations (e.g., via CrM supplementation or disease). For example, increases in myoblast fusion (shown in vitro [206] and in vivo [207]) and subsequent myotube growth after CrM administration [47] might involve the cellular mechanical energy properties and the optimization of cytoskeleton dynamics. Cr has a well-documented energy buffering effect [28]. Moreover, it has been shown that Cr enhances actin polymerization [175] and regulates scaffolding and motor proteins that control mechanosensing MAPKs [206,208]. This dynamic biosensor activity of CK/PCr system under the cellular allostasis model also provides a possible mechanistic basis as to why CrM supplementation favorably affects glucose management [126,209]. Specifically, the possible optimization of motor proteins (i.e., cellular mechanics) participating in the transport of GLUT4-containing vesicles to the plasma membrane (i.e., kinesins [KIF3 and KIF] and myosins [MYO5 and MYO1C]) and activation of energy-sensing signaling pathways due to the higher energy availability following CrM supplementation could facilitate improvements in glucose metabolism. This is supported by the fact that even though glucose tolerance is improved, several studies have failed to show a higher muscle content of GLUT-4 protein after CrM administration [209]. Additionally, cytolinker and motor proteins are important components that regulate signaling pathways like MAPKs [208], which in turn might trigger the IGF-I/Akt1/AS160 and/or the mTORC2/Akt1/AS160 pathways to promote GLUT-4 translocation [210,211,212]. This dynamic biosensor activity will be discussed in further detail according to the results of the convergent functional genomics analysis in an upcoming paper in this special issue. 

**Figure 3:**
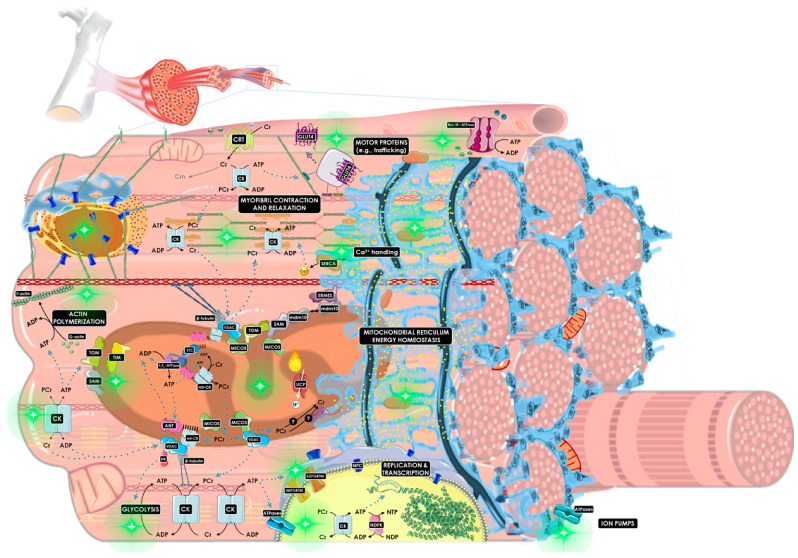
General overview of the CK/PCr system. The diagram represents the super-connected subcellular energy production and cellular mechanics of Cr metabolism. This chemo-mechanical energy transduction network involves structural and functional coupling of the mitochondrial reticulum (mitochondrial interactosome and oxidative metabolism), phosphagen and glycolytic system (extramitochondrial ATP production), the linker of nucleoskeleton and cytoskeleton complex (nesprins interaction with microtubules, actin polymerization, β-tubulins), motor proteins (e.g., myofibrillar ATPase machinery, vesicles transport), and ion pumps (e.g., SERCA, Na^+^/K^+^-ATPase). The cardiolipin-rich domain is represented by parallel black lines. Green sparkled circles represent the subcellular processes where the CK/PCr system is important for functionality (see the previous sections for rationale and citations). Several proteins of the endoplasmic reticulum–mitochondria organizing network (ERMIONE), the SERCA complex, the TIM/TOM complex, the MICOS complex, the linker of nucleoskeleton and cytoskeleton complex, and the architecture of sarcomere and cytoskeleton are not depicted for readability. ANT: adenine nucleotide translocase; CK: creatine kinase; Cr: creatine; Crn: creatinine; CRT: Na^+^/Cl^−^-dependent creatine transporter; ERMES: endoplasmic reticulum-mitochondria encounter structure; ETC: electron transport chain; GLUT-4: glucose transporter type 4; HK: hexokinase; mdm10: mitochondrial distribution and morphology protein 10; MICOS: mitochondrial contact site and cristae organizing system; NDPK: nucleoside-diphosphate kinase; NPC: nuclear pore complex; PCr: phosphocreatine; SAM: sorting and assembly machinery; SERCA: Sarco/Endoplasmic Reticulum Ca^2+^ ATPase; TIM: translocase of the inner membrane complex; TOM: translocase of the outer membrane complex; UCP: uncoupling protein; VDAC: voltage-dependent anion channel. Source: designed by the authors (D.A.B.) using figure templates developed by Servier Medical Art (Les Laboratoires Servier, Suresnes, France), licensed under a Creative Common Attribution 3.0 Generic License. http://smart.servier.com/ (accessed on 14 January 2021).

To summarize, the CK/PCr system can operate in a variety of capacities including: (i) acting as a spatio-temporal energy buffer (this would avoid the inactivation of ATPases and a net loss of adenine nucleotides by preventing the rise in intracellular ADP); (ii) preventing localized acidification through buffering [H^+^], which seems especially relevant in the early phase of physical exercise; (iii) becoming a source of increasing Pi at high work rates, which might reduce glycolytic activity; (iv) operating as a low-threshold ADP sensor that increases the thermodynamic efficiency of ATP hydrolysis. Finally, based on the model of predictive regulation [213], Cr metabolism should be seen as a noteworthy mechanism for cell survival and growth if we consider that the CK/PCr system behaves as a hub of chemo-mechanical energy transduction (i.e., dynamic biosensor) during a given allodynamic process. This complex balance of energy and mechanics may provide a manner to better understand the formation onset and progression of certain diseases and aging [204]. Figure 3 depicts a general overview of the CK/PCr system with the muscle cell as a model.

### 3.4. What Is the Role of Creatine among Tissues?

**Figure 4:**
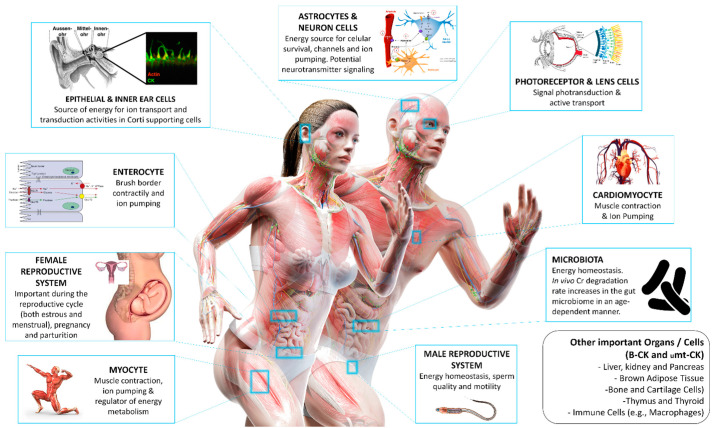
Importance of Cr metabolism in whole-body physiology. The CK/PCr system is essential for the chemo-mechanical energy transduction of cells/tissues with high, fluctuant, and constant energy demands. Source: designed by the authors (D.A.B.) using an anatomy template developed by 3dMediSphere (https://www.turbosquid.com/), licensed 3D standard Vray 3.60. accessed on 14 February 2020.

It has been mentioned that cytosolic and organelle-associated CKs constitute an intricate cellular energy buffering and transport system that connects PCr with sites of energy consumption, especially in tissues with high-energy needs. However, the function of the CK/PCr system as a chemo-mechanical energy transducer are different according to the biological process in non-muscle tissues. Table 2 summarizes the function of different CK isozymes according to the expression location. Additionally, Figure 4 summarizes the importance of CK/PCr system and Cr metabolism in tissues beyond skeletal muscle.

Given length restrictions, in-depth discussion of Cr metabolism in each tissue is not provided in-text. However, we aim to give particular attention to Cr metabolism and gut physiology given that this has been vastly understudied. Over 100 trillion microbes reside in the human intestine, and most are located in the colon. A high proportion of gut microbiota are bacteria, but it is notable that protozoans, fungi, archaea, and viruses might be also present. From an evolutionary point of view, these microbes fulfill relevant functions in human metabolism (e.g., vitamin production, fiber digestion, immune system regulation) [214]. Analyses of the collective genomes of these microbiota have led to intense interest regarding how the gut microbiome affects human physiology [215]. Relevant to this review, human Cr and Crn are important markers of microbiota given that they are also eliminated from the host by the action of intestinal microorganisms [8]. Additionally, underexpression of GAMT (rate-limiting step of Cr biosynthesis) can be linked to a colitis phenotype, among other conditions, although CrM administration in homozygous GAMT mutants may ameliorate the symptoms [216]. This illustrates the relevance of Cr in vivo for rapid replenishment of cytoplasmic ATP within colonic epithelial cells in the maintenance of the mucosal barrier after injury. It is also worth noting that Marcobal et al. [217] showed that fecal levels of Cr and Crn were elevated in germ-free versus wild-type mice, which is consistent with previous studies showing an increase of these molecules in biofluids of antibiotic-treated mice. In this way, low Cr concentrations might negatively impact mucosal barrier integrity, which postulates this metabolite as an early functional biomarker of inflammatory bowel disease [218]. Furthermore, Cr and Crn degradation has been shown to be heightened in the gut microbiomes of older mice compared to the middle-aged and younger mice [219]. Although research on the potential of gut microbiota in sports nutrition is in its infancy, it seems that Cr concentrations might be regulated by the microbiome which highlights the potential effects of CrM supplementation in this regard. This might be relevant if we consider the microbial diversity in elite athletes [220] and the effect of gut microbiota on GAA (an intermediary compound of the Cr synthesis) concentrations via guanidinoacetase [221]. 

**Table 2:** Creatine kinases and creatine among tissues.

Tissue	CK Isozyme	Function
Brain	BB-CK uMtCK	Supports brain cells energy production and buffers ATP and ion pumping during electrical activity in neurons [50]. Oral Cr supplementation has been shown to improve memory in healthy adults, and potential benefits for aging and stressed individuals have been described [222]. Additionally, Cr supplementation seems beneficial in reducing the severity or enhancing recovery from mild traumatic brain injury, but further studies are needed not only as a post-injury therapy but also as a neuroprotective agent in populations at high risk of mild traumatic brain injury [223].
Heart	MB-CK sMtCK	PCr provides about 80% of the energy needed for contraction and ion pumping, and about 20% of energy is transported into the cytoplasm via adenylate kinase and glycolytic phosphotransfer pathways [133,224]. MB-CK is an acute myocardial infarction marker [225].
Testes	BB-CK uMtCK	Energy production and ATP buffer at axoneme, where microtubules and dynein use direct energy for sperm motility [13,226]. Cr concentrations and CK activity are potential indicators of sperm quality [227].
Uterus	BB-CK uMtCK	Special attention should be paid to the increased Cr demand during pregnancy due to the important role of the PCr/CK system in the uterus and placenta for the maintenance and termination of gestation [34,228,229].
Sensory organs	BB-CK MM-CK MB-CK uMtCK sMtCK	Visual system: important role in phototransduction by providing energy for the visual cycle, maintaining high local ATP/ADP ratios and consuming H^+^ produced by ATPases located in the outer segment and, thereby, preventing acidification [230].
		Auditory system: MM-CK is located in the strial marginal cells and dark cells while BB-CK in the inner hair cells. High levels of CK are also found in the cochlea’s inner and outer phalangeal cells. This provides a source of energy for ion transport and transduction activities in the inner ear [231].
		Olfactory system: Olfactory sensory neurons express BB-CK in the cilia [232]. In large cells within the olfactory neuroepithelium and ventral spinal cord, differential compartmentation of CK isoforms has been evident, with B-CK localized primarily in cell nuclei, whereas uMtCK is present in the cell body (but not within nuclei). In olfactory bulb neuroepithelium, both isoforms are expressed in the middle zone of the germinal layer associated with DNA synthesis [233].
		Tactile and skin system: BB-CK co-expresses with low amounts of uMtCK in suprabasal layers of the epidermis (cell of hair follicles, sebaceous glands, and the subcutaneous panniculus carnosus muscle). MM-CK and sMtCK were restricted to panniculus carnosus [234]. Epidermal CK is very important for cellular energy metabolism and might decline under oxidative stress conditions, including skin-aging processes; interestingly, application of Cr to skin cells in vitro and in vivo can refuel these cells energetically, and, thus, protect them against free radical-induced cell damage [235].
		Gustatory system: crucial for optimal cell and motor development and function [236]. CK is also involved in the control of maturation and maintenance of myofibers in the distal tongue [237,238].
Intestines	BB-CK uMtCK	Distributed in the brush border web region, specifically at the contractile-ring myosin, to supply energy for contraction [239,240].
Miscellaneous	BB-CK MB-CK uMtCK	CK has been associated with the clotting cascade by means of thrombin receptor signaling [241]. The CK/PCr system has also been implicated in the function of the immune cells [126]. Finally, Cr metabolism has been implicated in UCP-independent thermogenesis in the brown and beige adipose tissue [129,242], and B-CK has been shown to be a key effector of the futile Cr cycle [243].

### 3.5. What Is the Basis of Creatine Transport?

The CRT (SLC6A8) is the solute carrier responsible for the 2Na^+^/Cl^−^-dependent co-transport of Cr into the cells. However, SLC16A12 has also been identified as a transporter of guanidino compounds (including Cr, Crn and GAA) that affect plasma, urinary and renal concentrations although its physiological function is unknown [244,245,246]. As previously mentioned, CRT has shown a high affinity to Cr in the plasmalemma but neither Crn nor PCr act as substrates. It has been shown that SLC6A8 also mediates the GAA transport, particularly in brain cells [247]. The main reason for this high substrate specificity is the separation by no more than 2–3 carbon atoms (4.5–5 Å) between the carboxyl group (to possibly interact with G73 and the Na^+^) and the guanidine group (to establish a hydrogen bond with C144), which suggests the presence of a dipole moment in the binding site that facilitates orientation and accommodation of the ligand molecules [248]. The most efficient competitive inhibitor on Cr transport is the β-guanidinopropionic acid [249]. In humans, the gene encoding CRT is located in chromosome Xq28, and this gene is made up of 3747 base pairs and 13 exons (GenBank Accession Number L31409–official symbol *SLC6A8*, also known as *CRT1*) [17]. Notably, the localization of the *SLC6A8* gene is in close proximity to genes responsible for several neuromuscular disorders [250]. *SLC6A10P* (also known as *CRT2*) is a pseudogene located in the 16p11.2 genomic region [251]. *SLC6A10P* contains ≈97% nucleotide sequence similarity to *SLC6A8*, but has been suggested to have an early stop codon [252]. Although there are reports of mRNA expression for the *SLC6A10P* in testes [253] and the brain [254], there is no evidence in publications or databases about its translation to a protein and additional information is needed about the functional effects of the respective transcribed RNA. Interestingly, microdeletions in 16p11.2 are one of the most common recurrent genomic disorders associated with autism [255]. Please refer to the following BioGPS ID for more details about gene expression patterns in different tissues: *SLC6A8*-6535, and *SLC6A10P*-386757.

Cr is transported into the muscle cells exclusively by CRT1. This protein consists of 635 amino acids (≈70.5 kDa) [256], it has 12 membrane-spanning domains with the N- and C- termini facing the cytoplasm, and it contains a large extracellular loop between the third and fourth transmembrane domains with sites for N-linked glycosylation [257]. The current literature suggests at least four isoforms of the CRT1 are transcribed from the *SLC6A8* gene by alternative splicing, and these include SLC6A8A, SLC6A8B, SLC6A8C and SLC6A8D. The first splice variant of the full-length SLC6A8A, called SLC6A8B, was identified by cloning and sequencing two cDNAs from a human hippocampal library with a rat CRT cDNA-specific probe. Compared to the fully homologous protein, the authors found a novel protein sequence with four different segments [258]. Prior to this report, González and Uhl [259] reported two different sequences of the SLC6A8 mRNA (4.0–4.3 and 2.2–3.0 kb) using Northern Blot analysis. Additionally, Guerrero-Ontiveros and Wallimann [260] found two polypeptides that were ≈70 kDa and ≈50 kDa with identical amino- and carboxy-terminal regions, which were linked to the variant of the full-length transcript due to alternative splicing. More recently, in an attempt to characterize the SLC6A8B mRNA and protein, Martínez-Muñoz et al. [261] identified a new splice variant called SLC6A8C that contained 270 amino acids (≈27.6 kDa) in humans and mice. Ndika and colleagues subsequently identified a new variant that was identical to SLC6A8C with the exception of an in-frame deletion of exon 9 in human and animal cells, and this protein (SLC6A8D) contained 224 amino acids (≈15 kDa) [262]. Interestingly, these authors also demonstrated that these splice variants (SLC6A8C and SLC6A8D), while lacking transport function, increased Cr transport through co-expression with the full-length CRT. Previous research has similarly shown that splice isoforms of the Na^+^/Cl^−^-dependent neurotransmitter transporter family may facilitate trafficking of full-length transporters [263].

While increasing Cl^−^ concentration significantly augments Cr influx in vitro [264], research has focused mainly on the Na^+^-dependent regulation. For example, a series of hormones that increase the sodium gradient across the muscle cell membrane (via Na^+^/K^+^-ATPase) influence the net Cr uptake into skeletal muscle cells in vivo and in vitro. It has been shown that insulin (at supraphysiological concentrations), insulin-like growth factor 1 (IGF-1), 3,3’,5-triiodothyronine, and certain catecholamines (noradrenaline, isoproterenol and clenbuterol) can stimulate Cr transport through membrane receptor activation mechanisms [250,265]. Tyrosine phosphorylation is a conserved mechanism for regulating the transport of neurotransmitters via SLC6 Na^+^-dependent transporters [266,267], and Cr uptake can also be affected by this mechanism. CRT has amino acid residues in the amino-terminal, carboxy-terminal and intracellular domains that can be phosphorylated by different kinases including the cAMP-dependent protein kinase (PKA) and the Ca^2+^ -dependent protein kinase (PKC) [17]. In addition, CRT is post-translationally modified and has two N-glycosylation sites, located in domains 3–4 and 11–12 in the extracellular space [268]. Phosphorylation and glycosylation might be important in the regulation of CRT activity and localization. Derave et al. [269] demonstrated that electrical stimulation of incubated rodent skeletal muscles stimulates rapid Cr transport possibly by endosomal translocation of CRT from an intracellular pool to the sarcolemma, rather than *de novo* protein synthesis. It is interesting to note that proteins that have been associated with regulation of CRT [270], such as the serine/threonine-protein kinases 1 and 3 (also known as serum and glucocorticoid-regulated kinases, SGK1/3), are activated upon H_2_O_2_ accumulation [271], which was observed after the electrical stimulation protocol of Derave et al. [269]. Other in vitro and animal studies have found that several kinases regulate CRT activity [14,268,272]. Additionally, Almeida et al. (2006) demonstrated in vitro that Cr is synthesized and taken up by central neurons and released by exocytosis depending on an action potential, which implies certain mechanisms of vesicular translocation are responsible for CRT localization [273]. This is supported by the fact that human and animal studies have shown that Cr saturation (by CrM supplementation) or depletion (by β-GPA administration) result in variations in the maximum rate of transporter activity (V_max_) rather than changes in the total CRT levels [274,275]. For instance, in cardiomyocytes, these changes in V_max_ correlate with CRT decreases in the cell surface fraction, indicating that changes in the cell surface are associated with the cellular responses to changes in Cr availability [268]. 

Finally, it is worth noting that congenital CRT deficiency is associated with autism, epilepsy, neurological defects, and intellectual disabilities [276,277]. This neurometabolic disorder is part of the Cr deficiency syndrome [52]. Thus, examining structural determinants of substrate binding in the CRT will provide a deeper understanding of the regulation of Cr uptake as well as novel therapeutic ligands [248,278]. For a more detailed coverage, both on human pathology and on their different in vivo models (KO and KI mice and rats), of the genetic conditions (AGAT, GAMT, and SLC6A8 deficiencies) of the Cr deficiency syndrome please refer to [18,22,279]. 

## 4. Limitations/Strengths and Future Directions

This review should be read in the light of various limitations/strengths. First, data from in vitro and in vivo animal models should be interpreted with caution given they might not fully reflect cellular behavior in humans. Second, we did not describe how Cr metabolism affects immunity, cancer, and certain conditions through lifespan (i.e., elderly, pregnancy) since these conditions extend beyond the main scope of this review and will be covered in other invited reviews of this book/special issue on “Creatine Supplementation for Health and Clinical Diseases”. This bioinformatics-assisted review should be seen as an up-and-coming method to address the lack of systematization in narrative reviews that aim to describe and analyze potential mechanisms of action. For example, besides cross-referencing the query results from several databases, we performed a clustering of CK-interacting proteins based on the Markov Cluster Algorithm using an open-source bioinformatics tool. This enriched the biological significance behind the Cr metabolism under a systems biology approach with experimentally-validated information that would be cumbersome to manually extract. The Research Division of the Dynamical Business & Science Society—DBSS International SAS is leading an initiative to develop and standardize the reporting guidelines of bioinformatics-assisted reviews.

Future studies about Cr metabolism should examine the implications of the CK/PCr system on thermogenic futile cycles considering the novel findings that have been reported regarding the role of AMPK in regulating the UCP-independent thermogenesis in white adipose tissue. Future research should also address the age-dependent changes that occur in the microbiome that cause higher Cr degradation rates in vivo, and whether this could be counteracted through CrM supplementation. More research is also needed to evaluate the effects of CrM supplementation during low-carbohydrate high fat diets [280] since preclinical evidence has revealed a suppression of the positive effects on muscle performance after CrM administration (by downregulation of the IGF1/Akt/mTOR pathway) during high-fat diet in rats [281]. It is also worth noting that dynamic simulations are important tools that can be used to predict how molecules potentially affect physiology. In this regard, new models could be developed considering the recent methodologies for kinetic analysis of the transphosphorylation reactions of the CK [282]. This allows testing and iteratively improving the prediction models before the experimental verification of systems perturbations might occur. 

## 5. Conclusions

Cr and PCr play an essential role in the optimal functioning of tissues with high and fluctuating energy demands (e.g., muscle, brain, and heart). Moreover, alterations in Cr and PCr concentrations produce marked functional changes that lead to various types of diseases (e.g., cancer or pathologies associated with Cr deficiency syndrome). After performing a comprehensive and bioinformatics-assisted review, and under the cellular allostasis paradigm, the current scientific evidence suggest that the CK/PCr system is physiologically essential for life (i.e., cell survival, growth, proliferation, differentiation, and migration/motility), and provides an evolutionary advantage for rapid and localized support of energy- and mechanical-dependent processes. In this sense, the CK/PCr system could be viewed as a dynamic biosensor of the cellular chemo-mechanical energy transduction, which may explain various positive benefits of CrM supplementation and cellular pathophysiology of the Cr deficiency syndrome. Given this centralized role of Cr metabolism in whole-body physiology, further studies are needed in order to further examine how Cr supplementation may affect other unidentified aspects of health and disease.

## Figures and Tables

**Table 1:** Characteristics of the creatine kinase isozymes.

Enzyme Name and Commission Number	Isozyme	Gene Name	Ensembl ID †	Gene Location	UniprotKB	Subunit Structure and PDB Entry	Cellular Location	Tissue Location *
Creatine kinase EC: 2.7.3.2	M-type	*CKM*	ENSG00000104879	Chromosome 19: 45,306,414–45,322,977 Reverse strand.	P06732	Dimer of identical or non-identical chains (1I0E)	Cytosol	Skeletal muscle & heart
	B-type	*CKB*	ENSG00000166165	Chromosome 14: 103,519,659–103,523,111 Reverse strand.	P12277	Dimer of identical or non-identical chains (3B6R)	Cytosol, dendrite, extracellular exosome, extracellular space, mitochondrion, myelin sheath, neuronal cell body and nucleus	Mainly brain, but also in testes, retina, bone, among several others
	U-Type	*CKMT1A*	ENSG00000223572	Chromosome 15: 43,692,886–43,699,222 Forward strand.	P12532	Octamer of four CKMT dimers (1QK1)	Mitochondrial inner membrane and Extracellular exosome	Brain, heart, brown adipose tissue, among several others
	S-type	*CKMT2*	ENSG00000131730	Chromosome 5: 81,233,285–81,266,397 Forward strand.	P17540	Octamer of four CKMT dimers (4Z9M)	Mitochondrial Inner Membrane	Mainly skeletal muscle

Data extracted from Ensembl, UniProtKB, PDB, and Gene Ontology. The heterodimer MB-CK exists mainly in heart. * For more details related to expression in different tissues or conditions (i.e., pathologies) visit BioGPS (http://biogps.org/), a database of gene expression profiles for human tissues [101], using the following ID numbers: *CKM*-1158; *CKB*-1152; *CKMT1A*-548596; and *CKMT2*-1160. † Use the cross-reference from Ensembl to BioGrid, IntAct, MINT or STRING databases in order to analyze protein–protein interactions. Many other bioinformatic tools are currently available. Databases/repositories were accessed on 11 November 2020.

## Data Availability

Not applicable.
